# Sequence Analysis of Six Blood Pressure Candidate Regions in 4,178 Individuals: The Cohorts for Heart and Aging Research in Genomic Epidemiology (CHARGE) Targeted Sequencing Study

**DOI:** 10.1371/journal.pone.0109155

**Published:** 2014-10-02

**Authors:** Alanna C. Morrison, Joshua C. Bis, Shih-Jen Hwang, Georg B. Ehret, Thomas Lumley, Kenneth Rice, Donna Muzny, Richard A. Gibbs, Eric Boerwinkle, Bruce M. Psaty, Aravinda Chakravarti, Daniel Levy

**Affiliations:** 1 University of Texas Health Science Center at Houston, Houston, Texas, United States of America; 2 Cardiovascular Health Research Unit, Department of Medicine, University of Washington, Seattle, Washington, United States of America; 3 Department of Epidemiology, University of Washington, Seattle, Washington, United States of America; 4 Department of Health Services, University of Washington, Seattle, Washington, United States of America; 5 The Framingham Heart Study, Framingham, Massachusetts, United States of America; 6 The Population Sciences Branch, National Heart, Lung, and Blood Institute, National Institutes of Health, Bethesda, Maryland, United States of America; 7 Department of Specialties of Medicine, Geneva University Hospitals, Geneva, Switzerland; 8 Department of Statistics, University of Auckland, Auckland, New Zealand; 9 Center for Complex Disease Genomics, Institute of Genetic Medicine, Johns Hopkins University School of Medicine, Baltimore, Maryland, United States of America; 10 Human Genome Sequencing Center, Baylor College of Medicine, Houston, Texas, United States of America; 11 Group Health Research Institute, Group Health Cooperative, Seattle, Washington, United States of America; Sapienza University of Rome, Italy

## Abstract

**Background:**

Genome-wide association studies (GWAS) identified multiple loci for blood pressure (BP) and hypertension. Six genes – *ATP2B1*, *CACNB2*, *CYP17A1*, *JAG1*, *PLEKHA7*, and *SH2B3* – were evaluated for sequence variation with large effects on systolic blood pressure (SBP), diastolic blood pressure (DBP), pulse pressure (PP), and mean arterial pressure (MAP).

**Methods and Results:**

Targeted genomic sequence was determined in 4,178 European ancestry participants from the Cohorts for Heart and Aging Research in Genomic Epidemiology (CHARGE) Consortium. Common variants (≥50 minor allele copies) were evaluated individually and rare variants (minor allele frequency, MAF≤1%) were aggregated by locus. 464 common variants were identified across the 6 genes. An upstream *CYP17A1* variant, rs11191416 (MAF = 0.09), was the most significant association for SBP (P = 0.0005); however the association was attenuated (P = 0.0469) after conditioning on the GWAS index single nucleotide polymorphism (SNP). A *PLEKHA7* intronic variant was the strongest DBP association (rs12806040, MAF = 0.007, P = 0.0006) and was not in LD (r^2^ = 0.01) with the GWAS SNP. A *CACNB2* intronic SNP, rs1571787, was the most significant association with PP (MAF = 0.27, P = 0.0003), but was not independent from the GWAS SNP (r^2^ = 0.34). Three variants (rs6163 and rs743572 in the *CYP17A1* region and rs112467382 in *PLEKHA7*) were associated with BP traits (P<0.001). Rare variation, aggregately assessed in the 6 regions, was not significantly associated with BP measures.

**Conclusion:**

Six targeted gene regions, previously identified by GWAS, did not harbor novel variation with large effects on BP in this sample.

## Introduction

Hypertension, defined as systolic blood pressure (SBP) ≥140 mm Hg or diastolic blood pressure (DBP) ≥90 mm Hg, is a major factor contributing to risk of coronary heart disease, cerebrovascular disease, and renal failure [1]. More than a billion individuals worldwide have hypertension [2]. Genome-wide association studies (GWAS) have led to an improved understanding of biological pathways underlying inter-individual variation in BP [3], [4]. In the largest GWAS to date, 29 independent loci were identified that significantly influence SBP and DBP levels and hypertension risk [4]. The variant alleles were common, each with small to modest effect sizes, generally less than 1 mm Hg per allele for SBP and 0.5 mm Hg for DBP. GWAS of pulse pressure (PP) and mean arterial pressure (MAP) have identified additional novel loci influencing these BP measures [5]. Despite these findings, the genetic architecture of hypertension remains incompletely understood. Among the loci identified by GWAS, only cytochrome P450 17A1 (*CYP17A1*) was previously known to harbor Mendelian rare variants with large effects on BP, specifically as a result of 17α-hydroxylase deficiency [6]. We hypothesized that resequencing of BP loci identified by prior GWAS would uncover novel genetic variation with large effects influencing BP measures. To that end, we sequenced 6 BP loci identified by GWAS [3], *ATP2B1*, *CACNB2*, *CYP17A1*, *JAG1*, *PLEKHA7*, and *SH2B3*, in 4,178 individuals of European ancestry, and searched for novel common and rare genome sequence variation with large effects on SBP, DBP, PP, and MAP.

## Methods

### Study samples and BP measurements

The 4,178 individuals of European ancestry (EA) evaluated in this study come from the Atherosclerosis Risk in Communities (ARIC) Study (n = 2,001), Cardiovascular Health Study (CHS, n = 1,128), and the Framingham Heart Study (FHS, n = 1,049) [7]. All ARIC, CHS and FHS subjects provided written, informed consent to participate in research protocols that were approved by the University of North Carolina at Chapel Hill, Chapel Hill, NC (ARIC), University of Washington, Seattle, WA (CHS), and Boston Medical Center, Boston, MA (FHS) institutional review boards. The ARIC [8], CHS [9], and FHS [10], [11] studies have been described elsewhere. For the current study, a subset of the original, offspring, and the third generation FHS cohort participants was included, but restricted to one individual per pedigree. For both CHS and ARIC, the data used in this study are from the first examination during which BP was measured using a standardized Hawksley random-zero mercury column sphygmomanometer. Three sequential recordings for SBP and DBP were obtained and the mean of the last two measurements was used in this analysis, discarding the first reading. In FHS, SBP and DBP measurements were taken as the mean of two physician readings using a mercury sphygmomanometer at the first examination cycle. In all cohorts, BP lowering medication use was recorded from the medication history or medication inventory. For individuals taking anti-hypertensive medication, untreated BP values were estimated by adding 15 mm Hg to measured SBP and 10 mm Hg to measured DBP [12], [13].

The CHARGE Targeted Sequencing Study implemented a case-cohort study design, in which both a random sample of participants (Cohort Random Sample) and participants with extreme values on 14 traits (Phenotype Groups) were selected from each of the three participating cohorts. For example, individuals were selected from the extremes of the standardized residuals of SBP and DBP after adjustment for age, age-squared, BMI, and study site if applicable. The regression was carried out stratified by gender and equal numbers of men and women were chosen for sequencing. Of the 200 individuals selected from the ∼1% extremes of the BP distribution, 194 had available targeted sequence data. Our analysis included individuals with BP measures that were selected on the basis of extremes of SBP and DBP (n = 194), those selected from other phenotype groups (n = 2,193), and from the Cohort Random Sample (n = 1,791) [14].

### Targeted regions for 6 BP loci

Genomic sequence was determined for targeted regions of 79 kb at *ATP2B1*, 24 kb at *CACNB2*, 31 kb at *CYP17A1*, 41 kb at *JAG1*, 24 kb at *PLEKHA7*, and 16 kb at *SH2B3*. The targeted loci were selected from among all BP loci implicated by GWAS [3], [4], [5], based on biologic plausibility and strength of statistical evidence. Thus, we included a locus with a role in 17α-hydroxylase deficiency leading to a Mendelian form of hypertension (*CYP17A1*), a locus with a potential role in intracellular signaling (*PLEKHA7*), a locus with two independent genome-wide significant signals (*CACNB2*), and loci with evidence for pleiotropic effects with other cardiovascular traits (*ATP2B1*, *JAG1*, and *SH2B3*) [14]. Target selection was accomplished by downloading the sequence of the target from the University of California at Santa Cruz (UCSC) Genome Browser and selecting the transcript with the most exons and longest exons. These transcripts are NM_001001323 for *ATP2B1*, NM_201571 for *CACNB2*, NM_000102 for *CYP17A1*, NM_175058 for *PLEKHA7*, and NM_005475 for *SH2B3*. *JAG1*, also identified in GWAS for BP [4], was selected for the CHARGE Targeted Sequencing Study by a group working on musculoskeletal phenotypes [14]. The following elements were included in the targeted sequencing capture: exons (±20 bp flanking), 5′-UTR (adding 1 kb proximal), 3′-UTR (adding 1 kb distal), any high linkage disequilibrium (LD) region around the GWAS index SNP (r^2^≥0.9 based on HapMap SNPs) after excluding regions or high and low GC-content and also L1 or L2 or ALU repeats, and conserved elements in introns after excluding regions of high GC-content. The customized targeted sequencing methodology and quality control measures are described elsewhere [14].

### Statistical analysis of common variants

Blood pressure values were adjusted for age, age-squared, sex, and BMI using linear regression. Common variants were defined as variants with at least 50 copies of the minor allele, and were evaluated individually. Unweighted linear regression models with robust standard error estimates were conducted in ARIC and CHS, and a linear mixed effects model was utilized in FHS to account for family relatedness. Weighted analyses that accounted for the sampling probabilities in the study design were also performed (Lumley T, et al. http://stattech.wordpress.fos.auckland.ac.nz/files/2012/05/design-paper.pdf).

Additionally, we conducted conditional analyses for the 4 blood pressure traits. The phenotype used for the conditional analyses was the trait residual generated from a model that included all of the index SNPs identified in GWAS from each of the loci of interest: rs17249754 (*ATP2B1*), rs4373814 (5′ of *CACNB2*), rs1813353 (3′ of *CACNB2*), rs11191548 (*CYP17A1*), rs1327235 (*JAG1*) and rs381815 (*PLEKHA7*). Genotypes of the index GWAS SNPs were either obtained from existing GWAS data for the study participants, or were available from the sequence data.

Summary statistics from the three cohorts were meta-analyzed using an inverse variance approach [14]. P-values are reported from the unweighted analyses, and unbiased estimates of the magnitude of effects from the weighted analyses (Lumley T, et al. http://stattech.wordpress.fos.auckland.ac.nz/files/2012/05/design-paper.pdf). In order to account for multiple comparisons, we applied a Bonferroni correction for the number of variants analyzed. A total of 464 variants were observed within the 6 genes of interest resulting in a significance threshold of P<0.0001 (0.05/464).

### Statistical analysis of rare variants

We defined rare variants as those with MAF≤1%. For testing, we considered all rare variants as well as a subset restricted to annotated exonic categories (nonsynonymous, splicing, stop/gain, and synonymous). The aggregate contribution of rare variation in the 6 targeted loci was assessed by burden tests that collapse the count of alternate alleles (T1). We also used a modified version of the Sequence Kernel Association Test (SKAT) to take into account rare variants with different directions of association [14]. Results were meta-analyzed across the ARIC, CHS, and FHS cohorts by inverse-variance weighting using METAL [15]. Given 6 genetic regions, a significance threshold of P<0.008 (0.05/6) was used for the rare variant tests.

## Results

A total of 4,231 individuals were sequenced, of whom 4,178 had BP measures. Table 1 shows the characteristics of the study sample. A total of 464 common variants were identified across the 6 genes, including 6 common nonsynonymous variants and no common loss of function variants (Table 2). Table 3 shows the total number of rare variants (MAF<1%) observed in each of the cohorts for each targeted region. Of the annotated exonic rare variants observed in the targeted regions (Table 3), there was 1 stopgain and 1 splicing variant in *CACNB2*, 1 stopgain in *CYP17A1*, 2 stopgain variants in *JAG1*, and 1 stopgain and 1 splicing variant in *PLEKHA7*.

**Table 1:** Characteristics of the study participants.

Characteristic	ARIC (n = 2,001)	CHS (n = 1,128)	FHS (n = 1,049)
	mean ± SD	mean ± SD	mean ± SD
SBP (mm Hg)	120±19	136±23	121±17
DBP (mm Hg)	72±12	71±12	78±11
PP* (mm Hg)	48±14	65±19	43±10
MAP† (mm Hg)	88±13	92±14	93±13
Age (years)	55±6	72±5	38±9
BMI (kg/m^2^)	28±6	27±5	26±6
Male (%)	51	46	52

*PP calculated as SBP-DBP.

† MAP calculated as (DBP+(SBP−DBP)/3).

All BP measures in the table are not adjusted for use of anti-hypertensive medication.

**Table 2:** Characteristics of common variants identified across 6 blood pressure loci.

Function	*ATP2B1*	*CACNB2*	*CYP17A1*	*JAG1*	*PLEKHA7*	*SH2B3*	Total
Intronic	55	77	15	98	66	10	321
Intergenic	54	0	122	9	0	0	75
Synonymous	2	3	2	7	3	0	17
ncRNA_intronic	0	0	14	0	0	0	14
UTR3	0	2	0	4	3	2	11
Upstream	2	0	3	0	1	0	6
UTR5	0	4	1	0	0	0	5
Nonsynonymous	0	1	0	1	2	1	5
ncRNA_UTR3	0	0	5	0	0	0	5
Downstream	0	1	0	2	1	0	4
Nonsynonymous; splice	0	0	0	0	1	0	1
Total number of variants	113	88	52	121	77	13	464

**Table 3:** Observed rare variants across cohorts.

Cohort	*ATP2B1*	*CACNB2*	*CYP17A1*	*JAG1*	*PLEKHA7*	*SH2B3*
	Total (Functional variants*)					
ARIC	1085 (20)	448 (24)	365 (20)	616 (31)	428 (39)	140 (14)
CHS	641 (7)	310 (20)	215 (8)	368 (13)	235 (20)	92 (7)
FHS	621 (7)	298 (9)	122 (10)	416 (18)	274 (26)	104 (12)

*Number of exonic variants belonging to the following annotated categories: nonsynonymous, splicing, stopgain, or synonymous.

**Figure 1:**
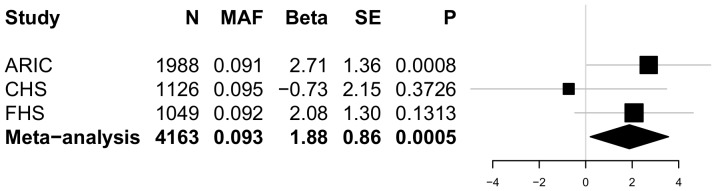
Forest plot for rs11191416 in relation to SBP. The beta estimate and standard error are reported from the unconditional weighted analyses. P-values are reported from unconditional unweighted analyses.

**Figure 2:**
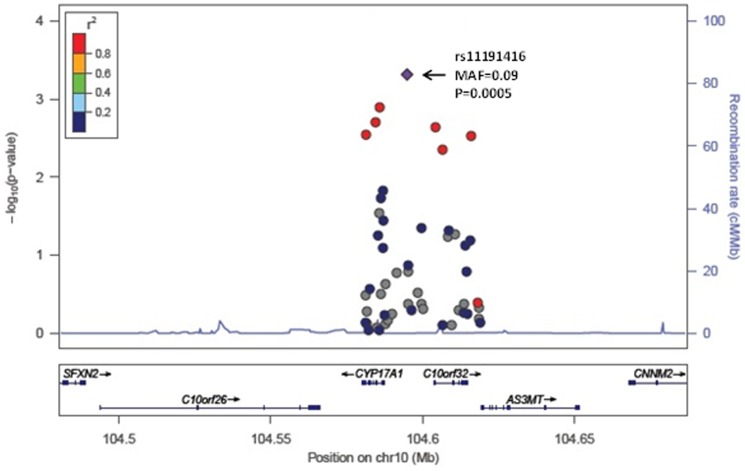
Regional association plot for *CYP17A1* in relation to SBP. Each circle represents a single variant. The triangle denotes the index variant. The color coding corresponds to the correlation (r^2^) between index variant and the other variants.

**Figure 3:**
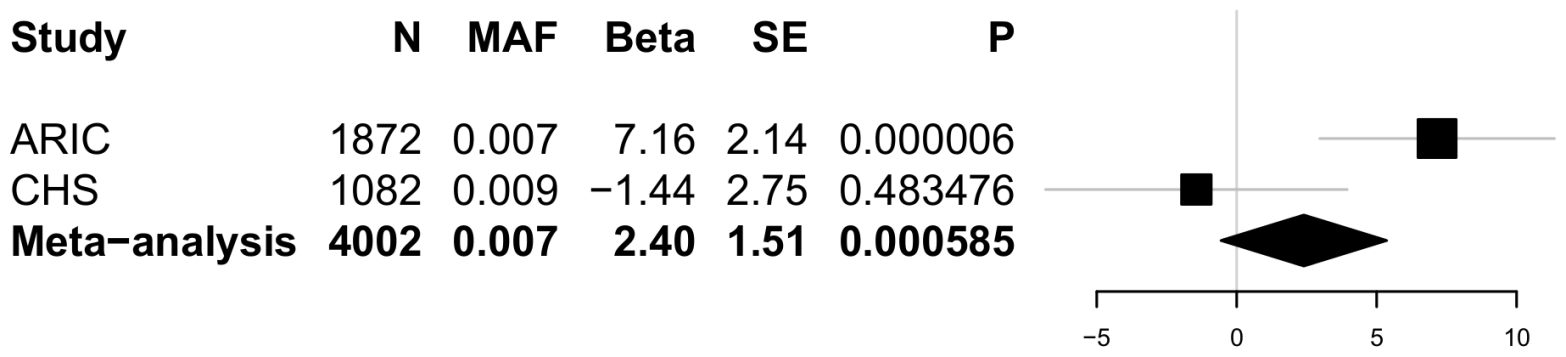
Forest plot for rs12806040 in relation to DBP. The beta estimate and standard error are reported from the unconditional weighted analyses. P-values are reported from unconditional unweighted analyses.

**Figure 4:**
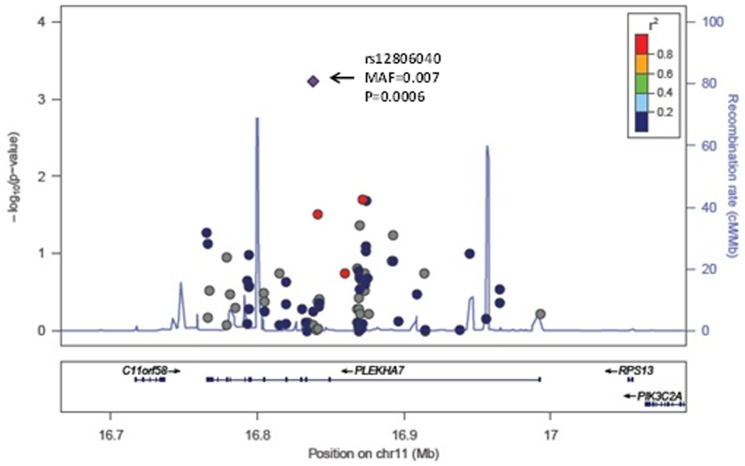
Regional association plot for *PLEKHA7* in relation to DBP. Each circle represents a single variant. The triangle denotes the index variant. The color coding corresponds to the correlation (r^2^) between index variant and the other variants.

None of the P-values for association of the common variants with the four BP traits reached our statistical significance threshold of P<0.0001. Table 4 summarizes the results for variants associated at P≤0.001 with any of the 4 BP traits. The most significant association with SBP was rs11191416 (MAF = 0.09, beta = 1.9 mm Hg, P = 0.0005) upstream of *CYP17A1* and in modest linkage disequilibrium (LD; r^2^ = 0.42) with the GWAS index SNP, rs11191548, located 241 kb away. The effect size estimate across cohorts for the relationship between rs11191416 and SBP is shown in the forest plot (Figure 1). The regional association plot for *CYP17A1* is shown in Figure 2. This same variant was also associated with PP (beta = 1.08 mm Hg, P = 0.0008). For DBP, the strongest association was with the intronic variant rs12806040 (MAF = 0.007, beta = 2.4 mm Hg, P = 0.0006) in *PLEKHA7*, that was not in LD (r^2^ = 0.01) with the GWAS index SNP rs381815 located 21 kb away. The forest plot in Figure 3 shows the significant positive association observed only in the ARIC study. The regional association plot for *PLEKHA7* is shown in Figure 4. An intronic SNP in *CACNB2*, rs1571787, was associated with PP (MAF = 0.27, beta = 1.02 mm Hg, P = 0.0003) and was in low LD (r^2^ = 0.34) with the 5′ GWAS index SNP, rs4373814, 14 kb away. In addition to these three SNPs, three variants (rs6163 and rs743572 in the *CYP17A1* region and rs112467382 in *PLEKHA7*) were associated with PP (P<0.001).

**Table 4:** Summary of meta-analysis results for common variants associated with blood pressure traits with P-value<0.001.

			Unconditional analyses			Conditional Analyses						
Trait	Chromosome: Position	MAF*	Beta	SE	P-value	Beta	SE	P-value	Function	Gene	rs number	Effect in ARIC/CHS/FHS
SBP	chr10:104594906	0.093	1.88	0.86	0.0005	0.12	0.83	0.0469	intergenic	*CYP17A1*	rs11191416	+−+
DBP	chr11:16837963	0.007	2.40	1.51	0.0006	2.30	1.46	0.0087	intronic	*PLEKHA7*	rs12806040	+−NA
PP	chr10:18473992	0.273	1.02	0.37	0.0003	0.60	0.36	0.0075	intronic	*CACNB2*	rs1571787	+−+
PP	chr10:104586914	0.382	−1.16	0.34	0.0005	−0.92	0.33	0.0041	synonymous	*CYP17A1*	rs6163	−−−
PP	chr10:104587142	0.364	1.24	0.34	0.0006	1.02	0.34	0.0040	UTR5	*CYP17A1*	rs743572	+++
PP	chr10:104594906	0.093	1.08	0.56	0.0008	0.27	0.55	0.0141	intergenic	*CYP17A1*	rs11191416	+++
PP	chr11:16914164	0.016	2.63	1.22	0.0009	2.94	1.24	0.0005	intronic	*PLEKHA7*	rs112467382	+−+

The beta estimate and standard error are reported from meta-analysis of results from weighted analyses.

P-values are reported from meta-analysis unweighted analyses.

Direction of effect across contributing studies is shown for the unconditional analyses.

*MAF across all cohorts.

After conditioning on the top index polymorphisms from GWAS, the observed effect size largely remained on the same order of magnitude as in the unconditional analyses (Table 4). The exception was an attenuated association for SBP and PP for rs11191416 upstream of *CYP17A1* and in modest linkage disequilibrium (LD; r^2^ = 0.42) with the GWAS index SNP, rs11191548, that was included in the conditional model. The association for rs112467382 and PP was slightly more significant (beta = 2.9 mm Hg, P = 0.0005) in the conditional model.

Rare variation, assessed by burden testing or SKAT in the 6 gene regions, was not significantly associated with any of the BP measures. Similarly, no significant association was observed in the T1 or SKAT tests when rare variation was subset to annotated exonic categories.

## Discussion

This study aimed to identify novel variation with large effects on SBP, DBP, PP, or MAP within 6 targeted BP loci identified previously through GWAS [4], [5]. None of the P-values for association of the common variants with the four BP traits reached the predefined statistical significance threshold of P<0.0001; therefore, we report results for common variants associated with blood pressure traits with P<0.001. Specifically, a low frequency intronic variant, rs12806040, in *PLEKHA7* was associated with DBP (meta-analysis weighted beta = 2.4 mm Hg). This variant can be viewed as independent of the GWAS index SNP in *PLEKHA7* given that is not in LD (r^2^ = 0.01) and it remains the most significant association for DBP (beta = 2.3 mm Hg, P = 0.0087) after conditional analyses. However, the observed association was primarily due to a significant positive association (P<0.00001) observed in the ARIC study. An additional low frequency intronic variant, rs112467382, in *PLEKHA7* influenced PP and remained associated with PP in the conditional analysis model (beta = 2.9 mm Hg, P = 0.0005), but with opposite direction of effect in CHS compared to ARIC and FHS.

The role of *CYP17A1* in SBP and PP was substantiated through the identification of a low frequency SNP (rs11191416, MAF = 0.093) upstream of *CYP17A1* and in modest linkage disequilibrium (LD; r^2^ = 0.42) with the GWAS index SNP, rs11191548, located 241 kb away. Two additional SNPs, rs743572 and rs6163, in *CYP17A1* influenced PP (P<0.001). These two common *CYP17A1* SNPs appear to be associated with PP independent from rs11191548, the GWAS SNP. The Genotype-Tissue Expression project (GTEx, www.gtexportal.org) database showed that rs743572 and rs6163 are eQTLs for a neighboring gene, arsenic (+3 oxidation state) methyltransferase (*AS3MT*) based on expression profiles in tissue from the left ventricle, suggesting a potential functional role for these variants. Additional eQTL analysis was based on gene expression measurement in whole blood and genotyping of approximately 5,000 FHS participants. Of the six distinct SNPs shown in Table 4, three (rs11191416, rs743572, rs6163) are cis-eQTLs with false discovery rate (FDR) <0.001. None were significant trans-eQTLs. These results confirm that rs743572 and rs6163 are eQTLs for *AS3MT*, and provide additional evidence that rs1191416 is associated with expression of the neighboring gene, 5′-nucleotidase, cytosolic II (*NT5C2*).

Targeted sequencing of 6 loci identified in BP GWAS did not identify consistently associated novel variation with large effect sizes, independent of the original GWAS signals, in this sample of 4,178 individuals. Given the sample size (n = 4,178) and 464 tests (alpha = 0.0001), and assuming a normal distribution, we had 80% power to detect an effect size of ∼10 mm Hg SBP and ∼5 mm Hg DBP for an allele frequency of 0.01.

This study includes well-phenotyped individuals from three community-based cohorts and enrichment in the sampling design for individuals selected from the extremes of the BP distribution. The targeted gene approach has the advantage of minimizing the multiple testing burden. Our study included only participants of European ancestry due to the fact that the candidate regions were identified from GWAS of European ancestry individuals. A limitation of this study is the reduced sample size compared with the GWAS from which the 6 BP loci were identified. This study considered only the first examination during which BP was measured and future studies incorporating repeat measures of blood pressure are warranted.

Comprehensive resequencing of the regions surrounding loci identified by GWAS is often regarded as the next step to identify the responsible variants underlying the GWAS signal. In general terms, there are two competing hypotheses: 1) a single (or only a few) common variants with modest effects, or 2) a synthetic association resulting from the combined large effects of rare variants [16]. Our study of 4,178 individuals from three CHARGE cohorts did not identify novel common variants nor an aggregate contribution of rare variants associated with large effects on BP traits. We conclude that no single association study on its own can evaluate the relative contribution of common versus rare variants for complex traits, and the final result may likely be both trait and population dependent. Based on the experience reported here, and from the work of others reported elsewhere [17], we believe that the large number of noncoding variants observed when comprehensively sequencing the entire locus creates an obvious signal-to-noise challenge that may be remedied by better annotation of non-protein encoding variants. Improved annotation may be used to help filter or weight variants in statistical tests of association. Additionally, like the original GWAS findings themselves, improved insight is likely to emerge with larger and more diverse sample sizes. As sequencing costs decline and new technologies emerge, these challenges are likely to be met.
